# Anti-inflammatory Effect of Ghrelin in Lymphoblastoid Cell Lines From Children With Autism Spectrum Disorder

**DOI:** 10.3389/fpsyt.2019.00152

**Published:** 2019-03-26

**Authors:** Yasunori Yamashita, Manabu Makinodan, Michihiro Toritsuka, Takahira Yamauchi, Daisuke Ikawa, Sohei Kimoto, Takashi Komori, Ryohei Takada, Yoshinori Kayashima, Kaori Hamano-Iwasa, Masatsugu Tsujii, Hideo Matsuzaki, Toshifumi Kishimoto

**Affiliations:** ^1^Department of Psychiatry, Nara Medical University School of Medicine, Nara, Japan; ^2^Faculty of Sociology, Chukyo University, Toyota, Japan; ^3^Research Center for Child Mental Development, University of Fukui, Fukui, Japan; ^4^Department of Development of Functional Brain Activities, United Graduate School of Child Development, Osaka University, Kanazawa University, Hamamatsu University School of Medicine, Chiba University and University of Fukui, Fukui, Japan

**Keywords:** ghrelin, autism, lymphoblastoid cell line, cytokine, immune system

## Abstract

The gut hormone ghrelin has been implicated in a variety of functional roles in the central nervous system through the brain-gut axis, one of which is an anti-inflammatory effect. An aberrant brain-gut axis producing immune dysfunction has been implicated in the pathobiology of autism spectrum disorder (ASD), and elevated expression of inflammatory markers has been shown in blood and brain tissue from subjects with ASD. We hypothesized that ghrelin may mitigate this effect. Lymphoblastoid cell lines from typically developed children (TD-C) (*N* = 20) and children with ASD (ASD-C) (*N* = 20) were cultured with PBS or human ghrelin (0.01 μM) for 24 h, and mRNA expression levels of the inflammation-related molecules interleukin-1β (IL-1β), interleukin-6 (IL-6), tumor necrosis factor-α (TNF-α), and nuclear factor kappa B (NF-κB) were measured to examine the effects of ghrelin as an anti-inflammatory agent. Expression levels of TNF-α and NF-κB mRNA, but not IL-1β or IL-6, were significantly elevated in ASD-C compared to TD-C. Ghrelin showed a tendency to reduce the expression of TNF-α and NF-κB, but this was not statistically significant. Considering the heterogenous pathobiology of ASD, we examined the effects of ghrelin on TD-C and ASD-C with expression levels of TNF-α and NF-κB in the highest and lowest quartiles. We found that ghrelin markedly reduced mRNA expression of TNF-α and NF-κB s in ASD-C with highest-quartile expression, but there were no effects in ASD-C with lowest-quartile expression, TD-C with highest quartile expression, or TD-C with lowest quartile expression. Together, these findings suggest that ghrelin has potential as a novel therapeutic agent for ASD with inflammation and/or immune dysfunction.

## Introduction

Autism spectrum disorder is a group of complex neurobiological disorders with a common triad of abnormalities in socializing, communication, and repetitive behavior (1, 2). Autism is typically diagnosed from the presence of these symptoms before three years of age. Although genetic factors have been considered to play a pivotal role in the pathogenesis of ASD, recently emerging evidence suggests that ASD and other neurodevelopmental disorders are likely to be influenced by gene-environment interactions, indicating that environmental factors may have a greater than expected effect on the pathogenesis of ASD (3).

Among well-documented environmental factors, the complex relationship between the gut microorganism population and brain function known as the gut-brain axis (4) is one of the most interesting areas of current research. The gut-brain axis is controlled by several factors including the composition of the gut microbiome, metabolites, and the gut immune system. Multiple studies have revealed that the gut flora affects brain development and function including mental state. Interestingly, immune dysregulation, and aberrant inflammatory response are well-known as key mediators of ASD pathogenesis. For example, subjects with ASD have increased proinflammatory cytokines in blood and excessive microglial activation in multiple brain regions (5–12). Recently, it has been reported that the composition of microbiome-modulating immune factors is associated with ASD symptoms, although mechanistic details remain unclear (13, 14).

In view of this accumulating evidence, we sought to investigate the function of ghrelin on the pathobiology of ASD. Ghrelin, a 28-amino-acid peptide hormone, is mainly secreted from the stomach and modulates the gut-brain axis, promoting release of growth hormone (GH) from the anterior pituitary gland (15, 16). Intriguingly, several studies indicate that ghrelin has anti-inflammatory effects against T cells and macrophages *in vitro* (17, 18). It has also shown protective effects against experimental autoimmune encephalomyelitis (EAE) and sepsis in animal models *in vivo* (19, 20). In humans, several clinical trials of ghrelin have already been completed for heart disease, chronic respiratory failure, loss of body weight, and inflammation (21–24). In addition, studies of rodent hippocampi have shown that ghrelin is related with higher brain functions, including learning and memory, through modulation of synaptogenesis (25–27). Furthermore, the expression level of ghrelin in blood is lower in children with ASD (ASD-C) compared to typically developed children (TD-C) (28). Ghrelin therefore shows promise as an agent for mitigating ASD phenotypes. In this study we investigated the effects of ghrelin on the expression levels of pro-inflammatory cytokines in lymphoblastoid cell lines (LCLs) from ASD-C and TD-C *in vitro*.

## Materials and Methods

### Participants

All study participants and their legal guardians provided written informed consent prior to enrollment. Diagnosis of ASD was made by two experienced child psychiatrists based on criteria outlined in the Diagnostic and Statistical Manual of Mental Disorders (5th Edition) with clinical interviews. The Structured Clinical Interview for DSM-IV was conducted to scrutinize any personal or family history of past or present mental illness.

This study was approved by the appropriate ethics committees of the Nara Medical University (No. 1319-5) and was carried out in accordance with the Declaration of Helsinki.

### Cell Culture

Peripheral blood mononuclear cells (PBMCs) were isolated from venous whole blood collected from each participant. All LCLs were developed by infecting PBMCs with Epstein-Barr virus produced in the supernatant of cultured B95-8 cells. These LCL strains were cultured in LCL medium consisting of Roswell Park Memorial Institute (RPMI) 1640 medium (Sigma-Aldrich, St. Louis, MO, USA) supplemented with 10% fetal bovine serum, 1 × anti-mycotics/anti-biotics (Thermo Fisher Scientific Inc., Waltham, MA, USA) and 1 × L-glutamine (Nacalai tesque, Kyoto, Japan) at 37°C in 5% CO_2_ humidified air. Proliferated cell aggregates were passaged twice per week after seeding at a density of 200,000 cells/ml.

### Ghrelin Stimulation

0.1 nmol human acyl-ghrelin (Peptide Institute Inc., Osaka, Japan) was dissolved in 500 μL phosphate-buffered saline (PBS; Wako Pure Chemical Industries, Ltd., Osaka, Japan) for use in the cell culture experiments. Ghrelin stimulation was performed by adding the above ghrelin solution to LCL medium with a final concentration of 0.01 μM when LCL passaging. The concentration of ghrelin used was determined according to previous studies both *in vitro* and *in vivo*, ensuring that the concentration used was near-physiological (29, 30). Cells were collected after 24-h incubation for the purpose of qRT-PCR. An equivalent volume of PBS was used as a vehicle treatment.

### Quantitative Reverse Transcription-Polymerase Chain Reaction (qRT-PCR)

Total RNA was extracted from the LCL culture samples using the AllPrep DNA/RNA/Protein Mini Kit (Qiagen, Hilden, Germany) according to the manufacture's protocol. Quantity of RNA was determined by measuring the absorbance at 260 nm with a DU 730 spectrophotometer (Beckman Coulter Inc., Fullerton, CA, USA). First-strand cDNA was synthesized from total RNA using an iScript kit (Bio-Rad Laboratories, Hercules, CA, USA), and qRT-PCR was performed using SYBR Premix Ex Taq II (Tli RNaseH Plus, TAKARA BIO INC., Otsu, Shiga, Japan) with StepOne Plus real-time PCR systems (Thermo Fisher Scientific Inc., Waltham, MA, USA). Relative quantification of the expression levels of target genes was performed by the delta CT method, using two constitutively expressed genes as internal controls: β-Actin (ACTB) and Cyclophilin A (CyA). The primer sequences are shown in Table 1.

**Table 1:** Primer sequences for qRT-PCR analysis.

**Gene name**	**Forward primer**	**Reverse primer**
β-Actin (ACTB)	GATGTGGATCAGCAAGCA	AGAAAGGGTGTAACGCAACTA
Cyclophilin A (CyA)	GCAGACAAGGTCCCAAAG	GAAGTCACCACCCTGACAC
Interleukin 1beta (IL-1β)	CTGTCCTGCGTGTTGAAAGA	GAAGACAAATCGCTTTTCCA
Interleukin 6 (IL-6)	AGTGAGGAACAAGCCAGAGC	CAGGGGTGGTTATTGCATCT
Tumor Necrosis Factor alpha (TNF-β)	GGCAGTCAGATCATCTTCTCG	CAGCTTGAGGGTTTGCTACA
Nuclear Factor Kappa B subunit 1 (NF-κB1)	CACTGTGAGGATGGGATCTG	CCCCTTATACACGCCTCTGT
Nuclear Factor Kappa B subunit 2 (NF-κB2)	TCTCGAATGGACAAGACAGC	TGCCATCCATTCTCATCATC

### Statistical Analysis

All data are represented as mean +SEM unless otherwise noted in the figure legends. All statistical analyses were performed by GraphPad Prism 6 software (GraphPad Software Inc., San Diego, CA, USA). Gene expression levels were compared among groups using one-way ANOVA, with *post-hoc* Tukey tests. Differences were considered significant where *p* < 0.05.

## Results

**Figure 1:**
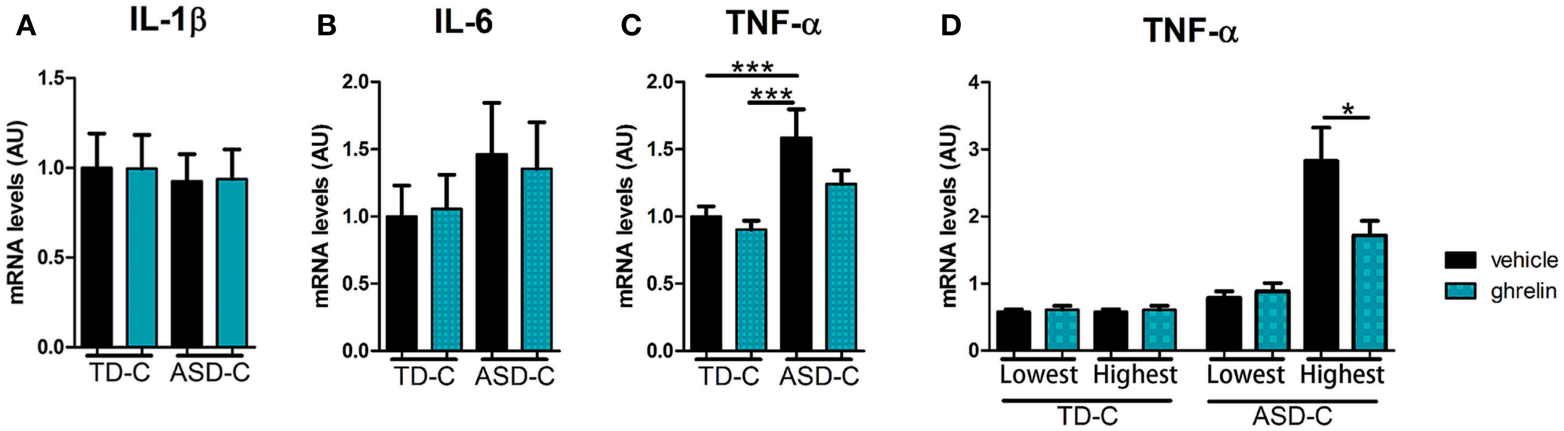
Ghrelin reduces mRNA expression of TNF-α in LCLs from ASD subjects with high, but not low, expression of TNF-α. **(A)** IL-1β mRNA expression in LCLs was similar between TD-vehicle, TD-ghrelin, ASD-vehicle, and ASD-ghrelin [*F*_(3, 76)_ = 0.0485, *p* = 0.986]. **(B)** IL-6 mRNA expression in LCLs was similar between TD-vehicle, TD-ghrelin, ASD-vehicle, and ASD-ghrelin [*F*_(3, 76)_ = 0.529, *p* = 0.664]. **(C)** TNF-α mRNA expression in LCLs was significantly different between TD-vehicle, TD-ghrelin, ASD-vehicle, and ASD-ghrelin [*F*_(3, 76)_ = 5.74, *p* = 0.0014]. TNF-α mRNA expression in ASD-vehicle was higher compared to either TD-vehicle or TD-ghrelin (*post-hoc* Tukey test: TD-vehicle vs. ASD-vehicle, *p* = 0.009; TD-ghrelin vs. ASD-ghrelin, *p* = 0.002). **(D)** Ghrelin reduced TNF-α mRNA expression in ASD-vehicle in the highest quartile of subjects for TNF-α expression, but not in the lowest quartile [*F*_(7, 32)_ = 13.6259, *p* = 5 × 10^−8^; *post-hoc* Tukey test: ASD-vehicle vs. ASD-ghrelin in the lowest quartile, *p* = 1.000; ASD-vehicle vs. ASD-ghrelin in the highest quartile, *p* = 0.011]. *P < 0.05, ***P < 0.001.

We established LCLs from 20 TD-C and 20 ASD-C. There were no significant differences in age or male/female ratio between the two groups (TD-C: age = 11.3 ± 2.7, M/F = 11/9; ASD-C: age = 11.5 ± 3.2, M/F = 13/7). In order to evaluate inflammatory markers in LCLs from ASD, and the anti-inflammatory effects of ghrelin, we measured mRNA expression levels of three proinflammatory cytokines (IL-1β, IL-6, and TNF-α) in LCLs from TD-C treated either with vehicle or ghrelin (TD-vehicle and TD-ghrelin, respectively) and from ASD LCLs treated with either vehicle or ghrelin (ASD-vehicle and ASD-ghrelin, respectively). Unexpectedly, the expression levels of IL-1β and IL-6 did not show any significant differences between TD-vehicle and ASD-vehicle [Figure 1A, IL-1β: *F*_(3, 76)_ = 0.0485, *p* = 0.986, and Figure 1B, IL-6: *F*_(3, 76)_ = 0.529, *p* = 0.664]. However, TNF-α expression was significantly increased in ASD-vehicle compared to TD-vehicle [Figure 1C, TNF-α: *F*_(3, 76)_ = 5.74, *p* = 0.0014; *post-hoc* Tukey test: *p* = 0.009]. While ghrelin treatment did not change the expression levels of IL-1β or IL-6 in LCLs from either TD-C or ASD-C [Figure 1A, IL-1β: *F*_(3, 76)_ = 0.0485, *p* = 0.986, and Figure 1B, IL-6: *F*_(3, 76)_ = 0.529, *p* = 0.664], TNF-α expression level had a non-significant tendency to be reduced by ghrelin in LCLs from ASD-C, but not from TD-C (Figure 1C, TNF-α, *post-hoc* Tukey test: TD-vehicle vs. TD-ghrelin, *p* = 0.947; ASD-vehicle vs. ASD-ghrelin, *p* = 0.224). Considering the heterogeneity of ASD (31), we hypothesized that only LCLs with either high or low expression of TNF-α might respond to ghrelin treatment. To address this question, we compared TNF-α mRNA levels in LCLs from the highest quartile for TNF-α expression (rank 1–5 of 20 children from each group) and those within the lowest quartile (rank 16–20 of 20 children from each group). We found that ghrelin markedly reduced TNF-α expression in the highest-quartile LCLs from ASD-C, but not in the lowest quartile LCLs [Figure 1D; *F*_(7, 32)_ = 13.6259, *p* = 5 × 10^−8^; *post-hoc* Tukey test: ASD-vehicle vs. ASD-ghrelin in the lowest quartile: *p* = 1.000; ASD-vehicle vs. ASD-ghrelin in the highest quartile: *p* = 0.011]. There was no significant difference in TNF-α expression within either the lowest- or highest-quartile LCLs from TD-C (Figure 1D, *post-hoc* Tukey test: TD-vehicle vs. TD-ghrelin in the lower quartile, *p* = 1.000, ASD-vehicle vs. ASD-ghrelin in the higher quartile, *p* = 0.948).

**Figure 2:**
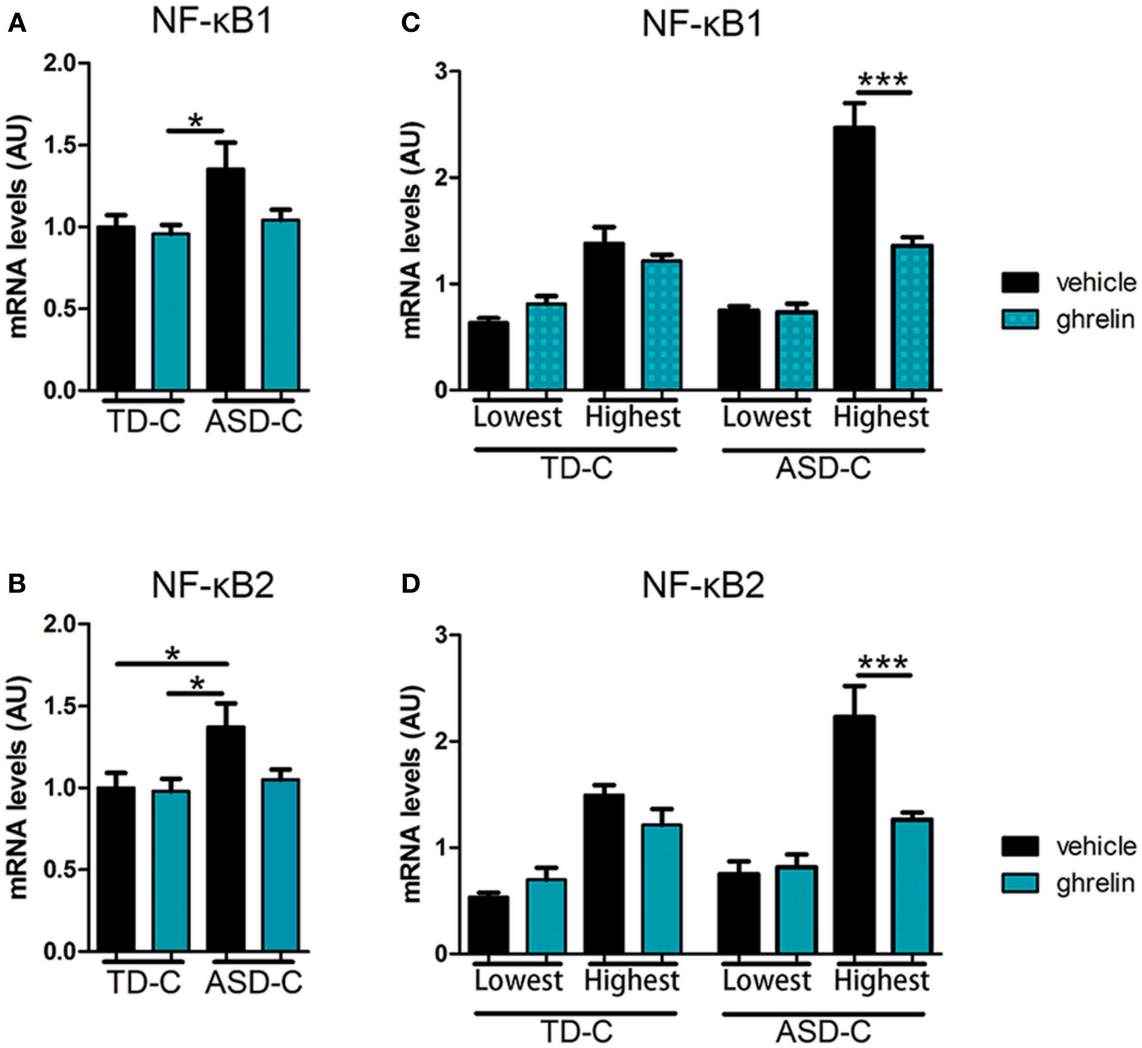
Ghrelin reduces mRNA expression of NF-κB1 and NF-κB2 in LCLs from ASD subjects with high, but not low, expression of NF-κB1 and NF-κB2. **(A)** NF-κB1 mRNA expression in LCLs was significantly different between TD-vehicle, TD-ghrelin, ASD-vehicle, and ASD-ghrelin [*F*_(3, 76)_ = 3.36008, *p* = 0.02305] and *post-hoc* testing indicated a tendency for NF-κB1 expression to increase in ASD-vehicle compared to TD-vehicle and a significant increase of NF-κB1 expression in ASD-vehicle compared to TD-ghrelin (Tukey test: *p* = 0.062, *p* = 0.023, respectively). **(B)** Expression of NF-κB2 mRNA in LCLs was significantly different between TD-vehicle, TD-ghrelin, ASD-vehicle, and ASD-ghrelin [*F*_(3, 76)_ = 3.53367, *p* = 0.01867] and *post-hoc* testing indicated a significant increase in NF-κB2 expression in ASD-vehicle compared to either TD-vehicle or TD-ghrelin (Tukey test: *p* = 0.042, *p* = 0.019, respectively). **(C,D)** Ghrelin reduced NF-κB1 and NF-κB2 mRNA expression in ASD-vehicle in subjects from the highest quartile, but from the lowest quartile of TNF-α expression [NF-κB1: *F* (7, 32) = 28.29225, *p* = 5.52 × 10^−12^; *post-hoc* Tukey test: ASD-vehicle vs. ASD-ghrelin in the lowest quartile, *p* = 1.000; ASD-vehicle vs. ASD-ghrelin in the highest quartile, *p* = 0.001; NF-κB2: *F* (7, 32) = 15.04981, *p* = 1.62 × 10^−8^; *post-hoc* Tukey test: ASD-vehicle vs. ASD-ghrelin in the highest quartile, *p* = 0.001; ASD-vehicle vs. ASD-ghrelin in the lowest quartile, *p* = 0.720]. *P < 0.05, ***P < 0.001.

On the basis of previous reports indicating an association between NF-κB and ASD (32, 33), we next measured mRNA expression levels of nuclear factor kappa B (NF-κB), a convergent mediator of cellular inflammatory pathways. We found that the expression level of NF-κB1, a subtype of NF-κB, was higher in LCLs from ASD-C (Figure 2A; *post-hoc* Tukey test: TD-vehicle vs. ASD-vehicle, *p* = 0.062, TD-ghrelin vs. ASD-vehicle, *p* = 0.028). Expression of another NF-κB subtype, NF-κB2, was also significantly elevated in LCLs from ASD-C compared to those from TD-C [Figure 2B: *F*_(3, 76)_ = 3.53367, *p* = 0.01867; *post-hoc* Tukey test: TD-vehicle vs. ASD-vehicle, *p* = 0.042, TD-ghrelin vs. ASD-vehicle, *p* = 0.019]. Ghrelin produced a non-significant tendency to lower NF-κB1 and NF-κB2 mRNA expression in LCLs from ASD-C, but not from TD-C (Figure 2A; NF-κB1, *post-hoc* Tukey test: TD-vehicle vs. TD-ghrelin, *p* = 0.989, ASD-vehicle vs. ASD-ghrelin, *p* = 0.122, and Figure 2B; NF-κB2, *post-hoc* Tukey test: TD-vehicle vs. TD-ghrelin, *p* = 0.999, ASD-vehicle vs. ASD-ghrelin, *p* = 0.100).

Similarly to our analysis of TNF-α mRNA expression, we also compared LCLs from the lowest and highest quartiles of NF-κB mRNA expression. Among the highest quartile of LCLs, ghrelin significantly reduced both NF-κB1 and NF-κB2 mRNA expression in ASD-C, but not in TD-C [Figure 2C; NF-κB1: *F*_(7, 32)_ = 28.29225, *p* = 5.52 × 10^−12^; *post-hoc* Tukey test: TD-vehicle vs. TD-ghrelin in the highest quartile, *p* = 0.966, ASD-vehicle vs. ASD-ghrelin in the highest quartile, *p* = 0.001, and Figure 2D, NF-κB2: *F*_(7, 32)_ = 15.04981, *p* = 1.62 × 10^−8^; *post-hoc* Tukey test: TD-vehicle vs. TD-ghrelin in the highest quartile, *p* = 0.853, ASD-vehicle vs. ASD-ghrelin in the highest quartile, *p* = 0.001]. There were no significant differences in NF-κB1 or NF-κB2 expression in the lowest-quartile LCLs from either TD or ASD (Figure 2C, NF-κB1, *post-hoc* Tukey test: TD-vehicle vs. TD-ghrelin in the lowest quartile, *p* = 0.992, ASD-vehicle vs. ASD-ghrelin in the lowest quartile, *p* = 1.000, and Figure 2D, NF-κB2, *post-hoc* Tukey test: TD-vehicle vs. TD-ghrelin in the lowest quartile, *p* = 0.492, ASD-vehicle vs. ASD-ghrelin in the lowest quartile, *p* = 0.720).

## Discussion

Unfortunately, there are no drugs to treat the three core symptoms of ASD: social challenge, communication difficulties, and repetitive behavior (34). Although a substantial number of researchers have made a strong effort to develop effective medicines for ASD treatment, only few drugs have been approved to ameliorate irritability in ASD subjects (35). Aberrant immune activation is a promising source of new therapeutic targets, since multiple lines of evidence have associated it with ASD symptoms (5, 8, 36–38). In this study, we showed elevated concentrations of the inflammation-related genes TNF-α and NF-κB in LCLs from ASD-C compared to TD-C.

Lymphoblastoid cell lines are nearly-immortalized cells, developed from peripheral B lymphocytes, which can reflect features of PBMCs even after *in vitro* subculture (39). Thus, our findings using LCLs could reflect the results found in PBMCs and postmortem brains. In our model, mRNA expression levels of TNF-α and NF-κB2 were significantly increased in ASD-C, though NF-κB1 expression did not reach statistical significance.

Mainly secreted by macrophages under pathogenic conditions, TNF-α triggers a variety of signaling pathways (40). It has been shown that TNF-α induces NF-κB activation and nuclear translocation through inhibition of κB, and also increases NF-κB transcription (41, 42). NF-κB is a protein complex that controls transcription of a number of genes, and plays a central role in the regulation of the immune response by producing inflammatory cytokines and chemokines such as IL-1b, IL-6, and TNF-α (43, 44). Young et al. reported an increase of extranuclear and nuclear-translocated NF-κB p65 expression in brain tissues of subjects with ASD compared to healthy controls, and this phenotype was expressed in neurons, astrocytes, and microglia (45). A significant increase in NF-κB DNA binding activity in peripheral blood samples of children with autism has also been found (32). Other immune factors associated with ASD include IL-17 receptor A, increased expression of which has been seen in monocytes of subjects with ASD, along with its activation by IL-17A-induced nitric oxide synthase (iNOS) expression via stimulation of the NF-κB pathway, contributing excess neuro-inflammation via oxidative stress (46). Since maternal activation of the IL-17a pathway causes ASD-like symptoms (47), immune activation including IL-17 and NF-κB increase risk of ASD. These findings warrant further investigation of the role of TNF-α/NF-κB signaling in ASD pathobiology. Another, more general, question for future investigation is which ASD symptoms are associated with which inflammatory markers.

Because ASD is a heterogenous disorder, it is possible that only a subgroup of ASD has immune activation. Indeed, our study demonstrated that the highest quartile of ASD subjects for TNF-α expression in LCLs virtually corresponded to the highest quartiles for NF-κB1 and NF-κB2 expression in LCLs (except for one person, data not shown), indicating that a subgroup of ASD had immune activation. Therefore, recent studies have sought to dissect the heterogeneity of ASD and design therapeutic approaches based on individual features of its subtypes (48). In this study, we also found some evidence supporting the possible therapeutic utility of ghrelin for the subgroup of ASD with inflammation and/or immune dysfunction. Together with the results of several clinical trials of ghrelin treatment demonstrating its safety for administration in humans (21–24), these findings encourage further studies to validate the effects of ghrelin using animal models of ASD with high inflammation and/or immune dysfunction such as BTBR mice and maternal immune activation-induced ASD models (49, 50). Since ghrelin signaling is implicated in anxiety and impairment of maternal care in rodents (51), which are often observed in subjects with ASD as comorbid symptoms, further investigation is warranted to evaluate which symptoms of ASD can be treated by ghrelin.

## Author Contributions

MM, MaT, and ToK supervised the overall research. YY, MiT, TY, DI, SK, TaK, RT, YK, and KH-I performed the experiments and collected the data. HM prepared materials and interpreted the data.

### Conflict of Interest Statement

The authors declare that the research was conducted in the absence of any commercial or financial relationships that could be construed as a potential conflict of interest.
